# Risk factors associated with short‐term mortality and recurrence of status epilepticus in dogs

**DOI:** 10.1111/jvim.16353

**Published:** 2022-01-07

**Authors:** Rory Fentem, Alberta de Stefani, Rodrigo Gutierrez Quintana, Emili Alcoverro, Gareth Michael Couper Jones, Pablo Amengual‐Batle, Rita Gonçalves

**Affiliations:** ^1^ Small Animal Teaching Hospital School of Veterinary Science, University of Liverpool Neston United Kingdom; ^2^ Queen Mother Hospital for Animals Royal Veterinary College, University of London Hertfordshire United Kingdom; ^3^ Small Animal Hospital School of Veterinary Medicine, University of Glasgow Glasgow United Kingdom; ^4^ Chestergates Veterinary Specialists Cheshire United Kingdom

**Keywords:** canine, epilepsy, outcome, seizures

## Abstract

**Background:**

Status epilepticus (SE) is an emergency associated with serious consequences for both patient and owner. Data regarding risk factors for short‐term mortality or recurrence in dogs with SE is limited.

**Objective:**

Identify risk factors associated with short‐term mortality (euthanasia or spontaneous death) and recurrence of SE in dogs.

**Animals:**

One hundred twenty‐four client‐owned dogs that sustained an episode of SE.

**Methods:**

Retrospective multicenter study using data collected from medical records of dogs presented in SE to the contributing institutions. Multivariable logistic regression analysis was performed using a manual backwards stepwise approach to identify risk factors associated with short‐term mortality and recurrence of SE after discharge.

**Results:**

Short‐term mortality for affected dogs was 29.8%. Factors significantly associated with short‐term mortality included increased patient age, shorter duration of hospitalization, development of SE before arrival, and SE caused by a potentially fatal etiology. Status epilepticus recurred in 27% of dogs that survived to discharge. Factors significantly associated with recurrence of SE included prior history of pharmacoresistant epilepsy and predominance of a focal seizure phenotype.

**Conclusions and Clinical Importance:**

Our results may be used to inform clinicians and dog owners regarding risk factors for both short‐term mortality and recurrence in dogs with SE.

**Abbreviations**
IE: idiopathic epilepsy
SE: status epilepticus

## 1 INTRODUCTION

Epilepsy is a complex brain disease characterized by an enduring predisposition to generate epileptic seizures, which manifest as a transient disturbance in any combination of motor, autonomic, cognitive, and sensory functions. In most cases, seizures are short, and their duration is typically fewer than 3 minutes.^1^ Status epilepticus (SE) is the result of failure of a seizure to spontaneously terminate. In current veterinary and human medical literature, SE is defined as either ≥5 minutes of continuous seizure activity or, in the case of generalized convulsive seizures, ≥2 discrete seizures between which incomplete recovery of consciousness occurs.^1^, ^2^, ^3^ In dogs, SE is a life‐threatening neurologic emergency that has both immediate and potentially long‐term implications for both the patient and owner.^4^, ^5^, ^6^ Status epilepticus may occur in patients with idiopathic epilepsy (IE), structural epilepsy, or be a reactive event (such as after intoxication or metabolic insults).^1^, ^7^ Rapid treatment of SE is imperative, given that increased duration of SE is associated with decreased likelihood of emergency treatment efficacy and increased risk of permanent neurologic and systemic morbidity.^4^, ^8^, ^9^, ^10^ Despite treatment, a substantial number of both human and veterinary patients with SE do not survive to hospital discharge.^11^, ^12^ Status epilepticus is frequently the initial manifestation of a seizure disorder in both dogs and humans, with a subset of patients going on to experience recurrent episodes of SE despite antiseizure drug therapy.^4^, ^7^, ^13^ Significant factors associated with recurrent SE in humans include age <4 years, female sex, lack of response to first‐line treatment of SE, and remote symptomatic (such as after a cerebrovascular accident or head trauma) and progressive (such as neurodegenerative diseases or malignancies not in remission) etiologies of SE.^14^ Although research has been performed regarding risk factors for the development of SE in dogs, little study has been undertaken to identify risk factors regarding mortality or recurrence of SE.^5^

Our aims were 2‐fold. We aimed to identify variables that significantly influence a dog's likelihood of mortality after an episode of SE. Second, in those that survived to discharge, we attempted to identify factors associated with recurrence of SE after discharge from the hospital.

## 2 MATERIALS AND METHODS

### 2.1 Case selection and inclusion criteria

Dogs admitted to the Small Animal Teaching Hospital (University of Liverpool), Queen Mother Hospital for Animals (Royal Veterinary College, University of London), and the Small Animal Hospital (University of Glasgow) that experienced SE in the hospitals between 2004 and 2017 were retrospectively identified from each hospital's database by searching for “status epilepticus.” Status epilepticus was defined according to the International Veterinary Epilepsy Task Force: ≥5 minutes of continuous seizure activity, or in the case of generalized convulsive seizures, ≥2 discrete seizures between which there is incomplete recovery of consciousness.^1^ Dogs were included in the study if they had experienced an episode of SE that resulted in hospitalization or sustained SE while hospitalized and had medical records available for review. Dogs were excluded from participation if their medical records were incomplete, if they received no treatment for SE or if a diagnosis was not recorded.

The following data were extracted from the patients' medical records: age, sex, breed, neuter status, body weight, clinical, and neurological examination findings on admission, age at first seizure episode, whether the dog had seizured previously or if SE was the first seizure episode identified, if initiation of SE was before admission or during hospitalization, underlying etiology for SE (and its potential to be considered fatal independent of SE), the presence of preexisting comorbidities (preexisting diseases not directly related to SE), acute complications during hospitalization for SE (pyrexia, acute kidney injury, acute liver injury, or aspiration pneumonia), a history of pharmacoresistant epilepsy (<50% decrease in seizure frequency with at least 2 antiseizure medications despite optimal dose, or serum concentration or both), predominant seizure phenotype (focal vs generalized), duration of first SE episode resulting in referral hospitalization (categorized as <30, 30‐60, or >60 minutes), duration of hospitalization, whether insured or not insured, whether dogs survived to discharge, neurologic status at discharge, and whether and when SE subsequently recurred after discharge in those that survived. Treatments administered to each patient were identified from the medical records. Many different treatment combinations were used and so each medication was assessed individually, but treatment protocols also were categorized as including constant rate infusions (CRIs) or not and, if so, of propofol or benzodiazepines. Initial response to benzodiazepine administration, even if it resulted in treatment failure minutes later also was recorded.

Possible underlying etiologies for SE were categorized as IE, structural epilepsy, or reactive seizures. Idiopathic epilepsy was diagnosed after exclusion of reactive seizure etiologies, along with cerebrospinal fluid analysis and either normal magnetic resonance imaging of the brain or abnormalities consistent with postictal brain pathology.^15^, ^16^ Structural epilepsy included vascular, neoplastic, inflammatory, traumatic, degenerative, or congenital diseases capable of contributing to epileptic seizure episodes, which were identified using some combination of cerebrospinal fluid analysis, magnetic resonance imaging, and necropsy examination. Reactive seizures were diagnosed if a metabolic etiology or intoxication was identified or strongly suspected based on historical information (such as exposure to a seizurogenic toxin, hepatic encephalopathy, hypertriglyceridemia, hypoglycemia, electrolyte disturbances, hyperthermia, or cerebral hypoxia). Intoxication was the designated etiology in dogs in which toxin exposure was confirmed or suspected based on witnessed ingestion of a known toxin. Metabolic seizure etiologies were established based on clinicopathological investigations and diagnostic imaging if deemed clinically appropriate.

An etiology was considered potentially fatal if it could cause death regardless of SE. This criterion was modified and applied from a human medical classification, and included large vessel ischemic and hemorrhagic stroke, acute central nervous system infections, severe systemic infection, brain neoplasia, chronic renal insufficiency requiring dialysis, systemic vasculitis, metabolic disturbance or acute intoxication, eclampsia, and intracranial tumor surgery.^17^ Noninfectious meningoencephalitis also was considered potentially fatal in our study.

For the purpose of our study, recurrence of SE while the patient was still hospitalized was not considered true recurrence but instead considered a treatment failure or inadequate treatment. For dogs in which SE recurred, the time between discharge and first recurrence was recorded. Dogs were discharged after at least 24 hours of being seizure‐free. Electroencephalographic confirmation of nonconvulsive seizure activity was not performed. All cases were managed by board‐certified or board‐eligible veterinary neurologists, or veterinary neurology residents under supervision. Ethical approval for use of data was granted by the Ethics Committee of the University of Liverpool (VREC522) and Royal Veterinary College, University of London (URN SR2019‐0324).

### 2.2 Statistical analysis

Statistical analysis was performed using the software SPSS 22.0 (SPSS Inc, Chicago, Illinois). Continuous data were tested for normality using the Shapiro‐Wilk test. Most data were not normally distributed, and therefore descriptive statistics were calculated as medians and interquartile ranges (IQR).

Univariable logistic regression was performed to identify clinical variables associated with short‐term mortality and with recurrence of SE. Any independent variable demonstrating some association on preliminary univariable analysis (*P* < .25) was considered for inclusion in a multivariable model. Before multivariable analysis, all variables were assessed for correlation using Spearman's rank correlation coefficients. If Spearman's rank correlation coefficient was >0.8, the most statistically significant or biologically plausible variable was selected. The goodness‐of‐fit of the final models was assessed using the Hosmer‐Lemeshow test.

Multivariable models then were constructed using a manual backwards stepwise removal approach. Variables with *P* < .05 were retained as statistically significant. Variables included in the univariable analysis for short‐term mortality included: age, sex, neuter status, body weight, institution, whether or not SE was the first seizure episode identified, if initiation of SE was before admission or during hospitalization, underlying etiology for SE (categorized as IE, structural epilepsy, or reactive seizures), if etiology was considered fatal independent of SE, presence of acute complications during hospitalization for SE (categorized as aspiration pneumonia, acute kidney injury, or acute liver injury), identification of pyrexia during hospitalization, history of pharmacoresistant epilepsy, identification of preexisting comorbidities, predominant seizure phenotype (categorized as focal or generalized), duration SE (categorized as <30, 30‐60, or >60 minutes), duration of hospitalization (days), insurance status, whether antiseizure medications were administered before the SE episode, which antiseizure medications were used during the SE episode (categorized as diazepam, midazolam, phenobarbital, levetiracetam, propofol, ketamine, and rectal potassium bromide), number of medications used during SE episode, initial response to benzodiazepine administration and whether CRIs of benzodiazepines or propofol were used during management of the SE episode. Institution was forced into the multivariable model because of the lack of a sample size power calculation that allowed complete rejection of the effect of this variable in order to control for it as a potential confounding variable.

Variables included in the univariable analysis for recurrence of SE included: age, sex, neuter status, body weight, institution, whether SE was the first seizure episode identified, underlying etiology for SE (categorized as IE, structural epilepsy, or reactive seizures), history of pharmacoresistant epilepsy, predominant seizure phenotype (categorized as focal or generalized) and whether new antiepileptic medications had been started after initial management of the SE episode. Finally, time‐to‐event analysis was used to construct a Kaplan‐Meier plot of cumulative probability of nonrecurrence.

## 3 RESULTS

### 3.1 Descriptive statistics

Of 175 cases initially identified from the database search, 124 cases fulfilled the inclusion criteria. Forty‐five cases were excluded because of incomplete medical records, 4 cases were excluded because a diagnosis could not be established, and 2 cases were excluded because treatment for SE was not attempted. Forty‐seven cases were contributed from the University of Liverpool, 28 from the Royal Veterinary College, University of London, and 49 from the University of Glasgow. The median age of dogs included was 54 months (IQR, 24‐96 months). Median body weight was 15.4 kg (IQR, 8.6‐24 kg) and 49 dogs were female (36 neutered) and 75 male (47 neutered). Among the 124 dogs included, 39 different breeds were represented. The most frequently documented breeds were Labrador Retrievers (n = 17), crossbreed (n = 17), Border Collie (n = 7), Boston Terrier (n = 5), English Springer Spaniel (n = 5), Boxer (n = 5), Pug (n = 5), and French Bulldog (n = 5). These breeds alone comprised 53.2% of the study population.

Nineteen dogs (15.3%) developed their first episode of SE while hospitalized, compared to 105 dogs (84.7%) that experienced SE before hospital admission. Status epilepticus was the first seizure manifestation in 56 dogs (43.5%). The suspected seizure etiology was available for 124 cases, with 50 being diagnosed with IE (40.3%), 47 with structural epilepsy (37.1%), and 27 with reactive seizures (22.6%). The structural epilepsy category consisted of 28 dogs with inflammatory disease (22.6%), 16 with neoplasia (12.9%), 1 with cerebrovascular disease, 1 with traumatic brain injury, and 1 with hydrocephalus. Of the dogs with suspected reactive seizures, SE was caused by confirmed metabolic disease in 11 cases (hepatic encephalopathy in 8 cases, hyperthermia with suspected cerebral hypoxia in 1 case [secondary to respiratory distress in a brachycephalic dog], hypertriglyceridemia in 1 case, and hypoglycemia secondary to insulinoma in 1 case, and caused by suspected intoxication in 16 cases (12.9%).

Information regarding provision of antiepileptic medication before SE was available for all 124 dogs. Forty‐two (33.9%) dogs were receiving antiepileptic medication before SE.

Twenty‐seven (21.8%) dogs received rectally administered diazepam before hospital admission, with 8 (29.6%) of these dogs experiencing cessation of seizure activity after its administration. The median dose of diazepam administered rectally at home was 1 mg/kg (IQR, 0.85‐1.13 mg/kg).

While hospitalized, 119 (96%) dogs received a benzodiazepine as part of their treatment for SE, with 82.3% receiving diazepam (IV or rectally) and 36.3% receiving IV midazolam (some dogs received both diazepam and midazolam). Observable seizure activity was stopped in 46 (38.7%) of those dogs after administration of benzodiazepines.

An infusion of either diazepam or midazolam was used in the management of SE in 25% of dogs. Phenobarbital was administered in 75.8% of dogs, levetiracetam in 51.6% of dogs, and rectally administered potassium bromide in 7.3% of dogs. General anesthesia was induced using propofol in 52.4% of dogs and pentobarbital in 0.8% of dogs. Other infusions administered included either medetomidine or dexmedetomidine in 1.6% of dogs and ketamine in 4.8% of dogs. During management of SE, 15 dogs (12.1%) received 1 medication, 34 dogs (29%) received 2 medications, 43 dogs (34.6%) received 3 medications, 28 dogs (22.5%) received 4 medications, and 4 dogs (3.2%) received 5 medications. After SE, 76 of the 87 dogs (87.3%) that survived to discharge received additional maintenance antiseizure medication. Median duration of hospitalization for the dogs in the study was 3 days (IQR, 2‐6 days).

Neurological examination on admission was documented in the medical records of all 124 dogs. Fifty‐six (45.2%) dogs were seizuring at the time of presentation. Ataxia or proprioceptive deficits were evident in 42 dogs (33.8%). Paresis was documented in 17 dogs (13.8%). An obtunded or stuporous mentation was noted in 58 dogs (46.8%). Twenty‐eight dogs (22.6%) had evidence of a visual deficit (decreased menace response, decreased pupillary light reflexes, or blindness). Four dogs (3.3%) displayed nystagmus and 6 dogs (4.8%) displayed circling behavior.

Neurological examination at the time of hospital discharge was clearly documented in the medical records of 81 of the 87 dogs that survived. Twenty‐three dogs (28.4%) displayed ataxia or proprioceptive deficits. Paresis was documented in 14 dogs (17.3%). Thirty‐two dogs (39.5%) were considered obtunded. Visual deficits were documented in 14 dogs (17.3%). Residual nystagmus was observed in 1 dog (1.2%). Three dogs (3.7%) exhibited circling behavior at the time of hospital discharge.

### 3.2 Short‐term mortality

Eighty‐seven dogs (70.2%) survived to hospital discharge. Thirty‐seven dogs (29.8%) did not survive, with 10 spontaneously dying (8%) and 27 being euthanized (21.8%) during their initial hospitalization. Of those euthanized, 8 were euthanized during SE and the remaining 19 were euthanized after poor recovery from SE. On univariable analysis, age, seizure etiology group, duration of hospitalization, potentially fatal etiology, SE as first seizure episode, presence of SE before arrival, administration of propofol, poor initial response to benzodiazepine administration and use of a CRI showed some evidence of association (*P* < .25) with short‐term mortality (Table S1). However, on multivariable analysis, only increasing patient age, shorter duration of hospitalization, onset of SE before arrival and SE being caused by a potentially fatal etiology remained significant factors (Table 1). Of the dogs that died or were euthanized, 8 had IE (of which SE was the first seizure in 1 dog), 21 had structural epilepsy (of which SE was the first seizure in 12 dogs) and 8 had reactive seizures (of which SE was the first seizure in 6 dogs). Of the dogs that survived to discharge, 42 had IE (of which SE was the first seizure in 9 dogs), 25 had structural epilepsy (of which SE was the first seizure in 10 dogs), and 20 had reactive seizures (of which SE was the first seizure in 4 dogs).

**TABLE 1:** Results of the multivariate analysis evaluating variables associated with short‐term mortality

Short‐term mortality	*P*‐value	OR	95% CI
Increasing age (months)	.01	1.017	1.004‐1.03
Potentially fatal etiology	.004	5.653	1.764‐18.12
Presence of SE before hospital admission	.001	0.036	0.004‐0.221
Duration of hospitalization (days)	<.001	0.568	0.425‐0.759

Abbreviations: 95% CI, 95% confidence interval; OR, odds ratio; SE, status epilepticus.

### 3.3 Status epilepticus recurrence

**FIGURE 1:**
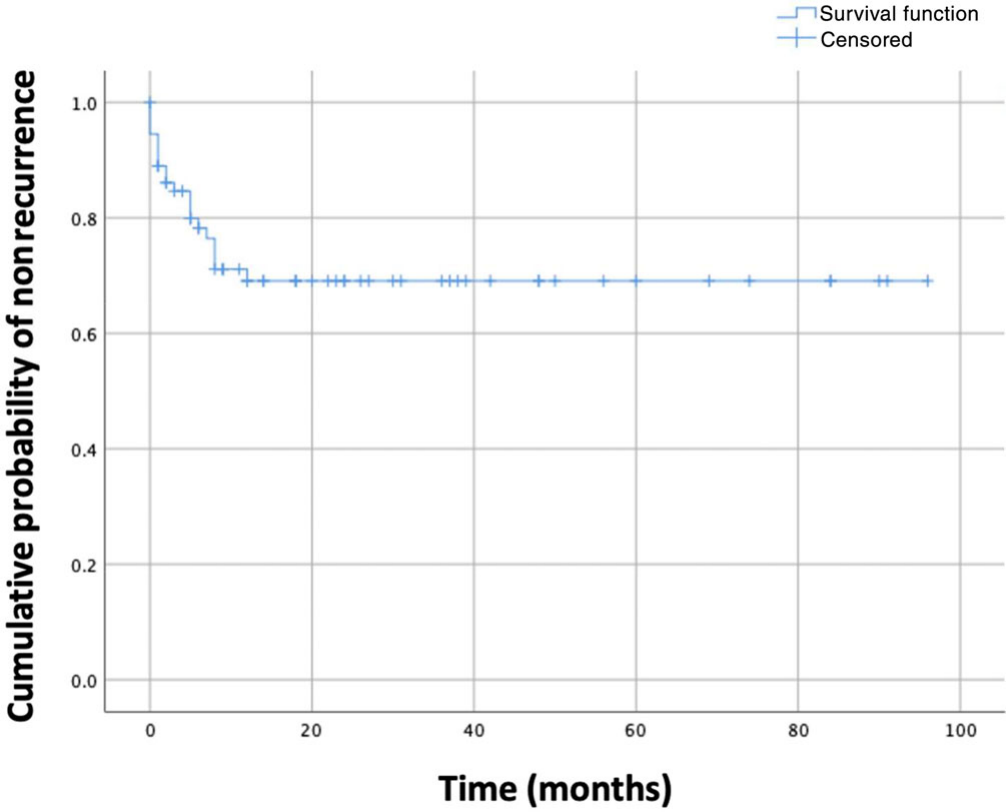
Kaplan–Meier estimate of nonrecurrence in dogs that survived a first episode of status epilepticus

Of the 87 dogs that survived to discharge, follow‐up information regarding SE recurrence was available for 74 dogs, with median follow‐up time of 9 months (range, 0‐96 months). Status epilepticus recurred in 20 of these dogs (27%; 95% confidence interval [95% CI], 17‐37%). Previous history of seizure episodes, SE as first seizure event, history of pharmacoresistant epilepsy, participating center, and type of seizures showed some evidence of association (*P* < .25) with recurrence of SE on univariable analysis (Table S2). On multivariable analysis, however, a history of pharmacoresistant epilepsy and type of seizures remained the only significant factors, with focal seizures associated with increased likelihood of SE recurrence (Table 2). A Kaplan‐Meier estimation of nonrecurrence is shown in Figure 1; median time to recurrence could not be estimated because recurrence occurred in <50% of cases. In the dogs in which recurrence was observed, 50% experienced recurrence within the first 2 months after hospital discharge, and all of those dogs exhibited recurrence of SE within 12 months after hospital discharge. Recurrence of SE was observed in dogs with IE, neoplasia, and inflammatory disease. No dogs with reactive seizures experienced recurrence of SE. Of the 20 dogs in which SE recurrence was observed, 13 had IE and 7 had SE.

**TABLE 2:** Results of the multivariate analysis evaluating variables associated with SE recurrence

SE recurrence	*P*‐value	OR	95% CI
History of pharmacoresistant epilepsy	.003	10.439	2.194‐49.681
Focal seizure type	.02	4.143	1.22‐14.072

Abbreviations: 95% CI, 95% confidence interval; OR, odds ratio; SE, status epilepticus.

## 4 DISCUSSION

The purpose of our study was to identify risk factors associated with short‐term mortality in dogs with SE and in those with SE recurrence. We found that short‐term mortality was clinically relevant in dogs with SE, with 29.8% of dogs dying or being euthanized during the hospitalization period in which the SE occurred. This finding is similar to that observed previously, where SE resulted in death or euthanasia in 38.4% of patients.^4^ A recent study examining risk factors associated with poor outcome in dogs with SE and cluster seizures reported in‐hospital short‐term mortality of 23%.^18^ Although this finding is not markedly dissimilar from our findings, the difference may be influenced by the fact that patients with SE comprised just 23% of the study population. Given that SE generally is regarded as more severe threat to life than cluster seizures, the different neurological consequences between the 2 conditions may account for this small difference, at least in part. Our result appears similar to those of human patients, in which SE had an associated short‐term mortality of up to 46% in specific populations.^19^ The in‐hospital mortality of our sample population generally was higher than that observed in studies of human patients with SE, with affected humans having a typical case fatality rate of approximately 15% as reported in a recent meta‐analysis.^20^ The short‐term mortality of our study and other veterinary clinical studies is skewed by the fact that many patients are euthanized as opposed to dying as a direct result of their SE, and the true case fatality rate may have been lower, and more similar to studies of human patients, should treatment have been continued. This possibility also may explain why an association was found with patients with increasing duration of hospitalization surviving to discharge. Those considered unlikely to survive were euthanized before lengthy hospitalization was undertaken, whereas those considered more likely to survive received ongoing medical treatment.

Etiologies considered potentially capable of fatality independent of SE were found to be significantly more likely to result in patient mortality. It is possible that this finding is related to these dogs carrying a generally worse actual or perceived prognosis that may have resulted in their prompt euthanasia, given that when etiologies were categorized into idiopathic, structural, or reactive epilepsy, this categorization was not found to be significantly associated with poor short‐term outcome. Increased patient age at the time of SE also was shown to be significantly associated with a higher likelihood of short‐term mortality in our population. It is possible that older dogs with SE are perceived as having a worse prognosis by their owners leading to treatment termination, or it may simply reflect the fact that owners may be less willing to invest time and financial resources in older dogs given the potential consequences of IE or SE.

Presence of SE before presentation to the referral hospital appears to be associated with decreased likelihood of short‐term mortality. The underlying reason for this finding is unclear from our data, given that this variable did not appear to be significantly associated with other variables such as underlying seizure etiology.

In our study, SE recurrence was observed in 27% of dogs that survived to hospital discharge. The likelihood of SE recurrence was significantly influenced by the patient having a history of drug‐resistant epilepsy and a history of focal seizures. The finding of focal seizures being markedly associated with SE recurrence is interesting. In humans with SE, the presence of focal seizures alone has been identified to impart a low likelihood of SE recurrence in comparison to other seizure types, regardless of etiology.^21^ When considering recurrence of seizure events, however, focal seizures generally are considered more challenging to treat than generalized seizures, in both human and animal epilepsy patients.^22^ It has been determined that the type of seizure exhibited by the patient should not be used in isolation to distinguish whether it is as the result of a structural lesion, reactive seizure episode or caused by IE. Focal epileptic events frequently are observed in dogs without structural causes of epileptic seizures.^2^ The underlying reason for focal seizures being associated with SE recurrence and seizure recurrence in general currently is unclear and additional studies will be required to identify whether this finding is repeatable.

It is not surprising that dogs with a prior history of pharmacoresistant epilepsy were significantly more likely to experience a recurrence of SE. Drug‐resistant epilepsy is a feature of approximately 30% of dogs with IE, with certain breeds being predisposed.^22^ The exact mechanisms of drug‐resistant epilepsy are unclear and may vary depending on each drug to which the patient is resistant, patient genetic factors and the nature of the epilepsy syndrome itself.^22^, ^23^, ^24^, ^25^ In humans, resistance to first‐line treatment of SE (typically benzodiazepines such as diazepam) has been associated with recurrence of SE, but the effect of an insufficient response to maintenance antiseizure medications has not been evaluated in predicting recurrence of SE.^21^

Kaplan–Meier analysis of our study population suggests that SE is most likely to recur shortly after the initial episode of SE, with approximately 50% recurrence within 2 months of the initial episode of SE. Although it appears to be a relatively infrequent event (occurring in just 27% of dogs in our study), owners should be made aware of the possibility of SE recurrence because of the emotional and financial costs associated with such an event. This initial period of potential SE recurrence likely reflects the more acute stages of brain injury that occur with SE, and the time required for antiseizure medications to reach peak therapeutic concentrations. With time, it appears that SE recurrence becomes less likely, and so treatment perseverance is important. Some patients, however, will continue to experience repeated episodes of SE despite provision of multiple antiseizure medications. A similar pattern of recurrence is observed in humans with SE in whom recurrence is observed in approximately one‐third of patients, and the majority of recurrence events appear to occur early in the follow‐up period.^21^, ^26^

A significant number of dogs in our study (43.5%) presented in SE as their first manifestation of a seizure disorder, as to 58% of dogs in another study.^4^ In our study population, previous focal seizures could not be excluded. This factor does not appear to affect a dog's short‐term prognosis or likelihood of SE recurrence, and owners should not be dissuaded from antiseizure treatment nor should dogs be euthanized simply because they have been presented in SE.

The population of dogs in our study contains a slight overrepresentation of male patients, which accounted for 60.5% of the study population. This finding is similar to that of a previous study in which a male overrepresentation also was identified.^4^ Nonetheless, patient sex was not significantly associated with either short‐term mortality or recurrence of SE in our study.

In common with many retrospective veterinary clinical studies, our study had some limitations. The retrospective identification of dogs with SE relied upon the quality of medical records kept at the time the patient was examined or last contact was made. As a result, some dogs that presented with SE were excluded because data was not readily extractable from their medical records. Imputation was utilized for some variables (eg, in cases in which an explicit duration of SE was not recorded). We attempted to accommodate for this factor by categorizing dogs based on SE duration, with seizure duration being estimated from the medical record. As previously mentioned, the fact that many of our dogs that died were euthanized makes assessment of actual short‐term mortality more challenging. Unfortunately, the reason for euthanasia was not clearly mentioned in the medical records of all dogs, which again makes identifying the reason for euthanasia difficult. The retrospective nature of our study makes evaluation of long‐term survival difficult and doing so was not attempted from this data set. Our incidence of SE recurrence appears generally to fit with that found in studies of human patients.^21^, ^26^ However, given a lack of long‐term follow‐up data in our dogs, the incidence of SE recurrence may in fact be higher. It is possible that some dogs were euthanized without reevaluation upon recurrence of SE. A prospective, longitudinal follow‐up study would allow for a more detailed assessment of long‐term outcome in patients with SE. The treatments delivered in our study population were not standardized and were at the discretion of the attending clinician. We attempted to accommodate for this variability by investigating treatments individually and by categorizing them. Lack of treatment standardization likely contributed to the observation that the treatments administered did not appear to substantially influence either short‐term mortality of likelihood of SE recurrence, as would otherwise be expected in this patient population.

In conclusion, our retrospective study on dogs with SE identified significant factors that influenced a patient's short‐term mortality (increased patient age, potentially fatal etiology, presence of SE before admission, and extended duration of hospitalization) and risk of SE recurrence (presence of focal seizures, history of pharmacoresistant epilepsy). Additional prospective studies are needed to confirm our findings and better characterize the long‐term outcome of dogs with SE.

## CONFLICT OF INTEREST DECLARATION

Authors declare no conflict of interest.

## OFF‐LABEL ANTIMICROBIAL DECLARATION

Authors declare no off‐label use of antimicrobials.

## INSTITUTIONAL ANIMAL CARE AND USE COMMITTEE (IACUC) OR OTHER APPROVAL DECLARATION

Ethical approval for use of data was granted by the Ethics Committee of the University of Liverpool (VREC522) and Royal Veterinary College, University of London (URN SR2019‐0324).

## HUMAN ETHICS APPROVAL DECLARATION

Authors declare human ethics approval was not needed for this study.

## Supporting information


**Table S1**. Univariable logistic regression results evaluating associations between different variables and short‐term mortality. Variables marked with an asterisk were subsequently used in the multivariable model.

**Table S2**. Univariable logistic regression results evaluating associations between different variables and recurrence of SE following discharge. Variables marked with an asterisk were subsequently used in the multivariable model.
