# Supplementary material for: Risk factors associated with short‐term mortality and recurrence of status epilepticus in dogs
Source: J Vet Intern Med. 2022 Jan 7;36(2):656–62. doi: 10.1111/jvim.16353 (PMC8965210; doi:10.1111/jvim.16353)
Supplement: Supplementary file 1 — Table S1. Univariable logistic regression results evaluating associations between different variables and short‐term mortality. Variables marked with an asterisk were subsequently used in the multivariable model. [file JVIM-36-656-s002.pdf]

Supplementary Table 1. Univariable logistic regression results evaluating associations between different variables and short-term mortality. Variables marked with an asterisk were subsequently used in the multivariable model.

Variable	*P*-value	Odds ratio	95% CI
**Bodyweight (kg)**	0.507	1.011	0.979 – 1.044
**Age (months)***	0.08	0.992	0.983 – 1.001
**Previous history of seizures**	0.4	1.577	0.738 – 3.37
**Gender**	0.634	0.83	0.386 – 1.185
**Neuter status**	0.414	1.389	0.631 – 3.057
**SE prior to admission***	0.242	1.592	0.731 – 3.465
**Benzodiazepines administered at home***	0.049	3.145	1.007 – 9.822
**Comorbidities**	0.933	0.968	0.453 – 2.069
**Pyrexia**	0.579	1.246	0.574 – 2.706
**Aspiration pneumonia**	0.543	0.751	0.299 – 1.888
**Acute kidney injury**	0.313	0.429	0.083 – 2.225
**Acute liver injury**	0.422	0.635	0.209 – 1.926
**Seizure etiology group***<br>**Idiopathic Epilepsy**<br>**Structural Epilepsy**<br>**Reactive seizures**	 (Ref)<br>0.211<br>0.211	 (Ref)<br>2.389<br>0.525	 (Ref)<br>0.776 – 7.35<br>0.192 – 1.439
**Potentially fatal etiology***	0.004	0.28	0.119 – 0.659
**SE duration**<br>**<30 mins**<br>**SE duration 30-60 mins**<br>**SE duration >60 mins**	 (Ref)<br>0.962<br>0.253	 (Ref)<br>0.977<br>1.697	 (Ref)<br>0.377 – 2.536<br>0.685 – 4.206
**SE before admission***	0.005	4.49	1.586 – 12.712
**SE as first known seizure***	0.157	0.577	0.269 – 1.236
**Predominant seizure phenotype**	0.856	0.923	0.386-2.204
**Availability of pet insurance**	0.331	1.684	0.589 – 4.812
**History of pharmacoresistant epilepsy***	0.135	4.935	0.609 – 39.981
**Antiepileptic medications used before SE***	0.045	2.486	1.022 – 6.045
**Number of antiseizure medications given during SE**	0.692	0.931	0.655 – 1.325
**Infusion used***	0.14	0.547	0.246 – 1.218
**Infusion type used:**<br>**Benzodiazepine**<br>**Propofol**<br>**Benzodiazepines and propofol**	 0.382<br>0.405<br>0.058	 0.538<br>0.681<br>0.342	 0.134 – 2.158<br>0.276 – 1.682<br>0.113 – 1.036
**Diazepam**	0.362	0.606	0.206 – 1.78
**Midazolam**	0.406	0.719	0.33 – 1.565
**Propofol***	0.17	0.583	0.27 – 1.26
**Ketamine**	0.897	0.892	0.156 – 5.085
**Phenobarbital**	0.897	0.943	0.386 – 2.301

**Levetiracetam**	0.941	0.972	0.457 – 2.069
**Rectal potassium bromide**	0.873	0.889	0.211 – 3.753
**Duration of hospitalisation***	0.01	1.233	1.052 – 1.446
**Centre**			
**Centre 1**	(Ref)	(Ref)	(Ref)
**Centre 2**	0.723	1.196	0.444 – 3.224
**Centre 3**	0.254	1.656	0.696 – 3.939
**Response to benzodiazepines***	0.027	2.649	1.115 – 6.293
