# Supplementary material for: Risk factors associated with short‐term mortality and recurrence of status epilepticus in dogs
Source: J Vet Intern Med. 2022 Jan 7;36(2):656–62. doi: 10.1111/jvim.16353 (PMC8965210; doi:10.1111/jvim.16353)
Supplement: Supplementary file 2 — Table S2. Univariable logistic regression results evaluating associations between different variables and recurrence of SE following discharge. Variables marked with an asterisk were subsequently used in the multivariable model. [file JVIM-36-656-s001.pdf]

Supplementary Table 2. Univariable logistic regression results evaluating associations between different variables and recurrence of SE following discharge. Variables marked with an asterisk were subsequently used in the multivariable model.

**Variable**	***P*-value**	**Odds ratio**	**95% CI**
**Bodyweight (kg)**	0.286	1.021	0.982 – 1.062
**Age (months)**	0.907	1.001	0.987 – 1.015
**Previous history of seizures***	0.013	7.2	1.518 – 34.139
**Gender**	0.703	1.228	0.428 – 3.526
**Neuter status**	0.271	2	0.583 – 6.864
**Identified comorbidities**	0.377	1.594	0.566 – 4.488
**SE etiologic category:**			
**Idiopathic epilepsy**	(Ref)	(Ref)	(Ref)
**Structural epilepsy**	0.795	0.862	0.28 – 2.648
**Reactive seizures**	0.998	0	0 – 0
**Potentially fatal etiology**	0.445	0.667	0.235 – 1.889
**Predominant seizure phenotype***	0.025	3.6	1.178-11
**History of pharmacoresistant epilepsy***	0.003	9.154	2.077 – 40.344
**Antiepileptic medications initiated following management of SE**	0.851	0.897	0.291 – 2.769
**Centre effect***			
**Centre 1**	(Ref)	(Ref)	(Ref)
**Centre 2**	0.025	0.15	0.028 – 0.791
**Centre 3**	0.108	0.387	0.122 – 1.232
**SE as 1st seizure episode identified***	0.105	0.364	0.107 – 1.235
